# Ire1 inhibitors attenuate *Candida albicans* pathogenicity and demonstrate potential for application in antifungal therapy

**DOI:** 10.3389/fmicb.2025.1648467

**Published:** 2025-09-03

**Authors:** Hua Wang, Mengyan Li, Qiuyue Wang, Huihai Zhao, Mengyu Jiang, Qi Cui, Daxin Lei, Keran Jia, Fukun Wang

**Affiliations:** Department of Clinical Laboratory, Bethune International Peace Hospital, Shijiazhuang, China

**Keywords:** *Candida albicans*, endoplasmic reticulum, Ire1, Ire1 inhibitor, 4**μ**8c, pathogenicity

## Abstract

**Introduction:**

*Candida albicans* is a common opportunistic pathogen responsible for both superficial and invasive infections. The unfolded protein response, triggered by endoplasmic reticulum stress, plays a crucial role in its survival and pathogenicity, with the endoplasmic reticulum stress sensor Ire1 serving as a key regulator. Pharmacological inhibition of Ire1 may therefore represent a novel antifungal strategy.

**Methods:**

We conducted molecular docking to identify small-molecule inhibitors targeting the RNase activity of *Candida albicans* Ire1, followed by in vitro assays assessing pathogenic traits and in vivo validation using a murine intestinal colonization model.

**Results:**

Three candidate inhibitors—MKC8866, STF083010, and 4μ8c—were predicted to interact with Ire1, but only 4μ8c exhibited consistent inhibitory activity. 4μ8c was found to significantly impair key pathogenic traits, including morphological transformation, adhesion, flocculation, and biofilm formation. Additionally, it enhanced the susceptibility of *Candida albicans* to antifungal drugs and reduced the expression of virulence-related genes. In vivo studies using a murine intestinal colonization model demonstrated that 4μ8c effectively reduced fungal colonization and intestinal tissue damage caused by *Candida albicans*.

**Discussion:**

These findings demonstrate that pharmacological targeting of the UPR pathway through Ire1 inhibition is feasible. 4μ8c emerges as a promising candidate that diminishes the adaptability and pathogenicity of *Candida albicans*, offering new insights into antifungal therapeutic development.

## 1 Introduction

In recent years, fungal infections have remained a significant threat to human health. It is estimated that billions of individuals contract fungal infections annually, leading to approximately 1.5 million deaths worldwide—comparable to the mortality rates of tuberculosis and malaria (Brown et al., 2012; Fisher et al., 2018; Pfaller and Diekema, 2010). Among fungal pathogens, *Candida* species are a major cause of both superficial and invasive infections, with *Candida albicans* (*C. albicans*) being the most prevalent species within this genus (Lamoth et al., 2018; Pappas et al., 2018). As an opportunistic pathogen, *C. albicans* can colonize mucosal surfaces of the gastrointestinal tract, genitourinary tract, oral cavity, and skin in a commensal relationship with the human host. However, under conditions such as immunosuppression, environmental alterations, or dysbiosis, *C. albicans* can overgrow, leading to superficial infections (e.g., oral thrush, vulvovaginal candidiasis) or invasive diseases (e.g., disseminated candidiasis) (Calderone and Fonzi, 2001; Nobile and Johnson, 2015).

The endoplasmic reticulum (ER) is a vital organelle in *C. albicans*, responsible not only for protein folding and trafficking but also for lipid biosynthesis and calcium homeostasis (Dominguez-Martin et al., 2018). Additionally, it plays a critical role in regulating cell wall composition and biofilm formation, which are essential for immune evasion and colonization (Douglas and Konopka, 2016; Malavazi et al., 2014). Thus, proper ER function is fundamental to the adaptability and pathogenic potential of *C. albicans*.

During host infection, *C. albicans* encounters various stressors, including nutrient deprivation, oxidative stress, antifungal agents, temperature fluctuations, and membrane damage (Brown et al., 2014; D'enfert et al., 2021). These conditions can disrupt ER homeostasis, leading to the accumulation of unfolded or misfolded proteins and triggering the ER stress (ERS) response. In response, cells initiate a series of adaptive mechanisms collectively termed the UPR to restore ER function (Hernandez-Elvira et al., 2018). Under transient or mild ER stress, the UPR enhances protein-folding capacity, suppresses protein translation, and activates autophagy, thereby promoting cellular survival. However, prolonged or severe ER stress can ultimately lead to apoptosis (Walter and Ron, 2011). In *C. albicans*, activation of the UPR is essential for survival within the host, as it mitigates immune stress and regulates the expression of virulence-related genes, thereby enhancing pathogenicity and dissemination. Therefore, the UPR signaling pathway plays a critical role in the symbiosis between *C. albicans* and the host, as well as in its pathogenicity.

In mammalian cells, three major ER stress sensors mediate the UPR: Ire1, ATF6, and PERK (Wiseman et al., 2022). Among these, the Ire1 pathway is the most evolutionarily conserved and is present in plants and fungi as well (Bashir et al., 2021; Li and Howell, 2021). In *C. albicans*, Ire1 is the sole ER stress sensor, mediating UPR activation and maintaining ER homeostasis (Sircaik et al., 2021). The Ire1 protein spans the ER membrane and consists of a sensing domain, a kinase domain, and a ribonuclease (RNase) domain. Ire1 signaling relies on the cooperative function of its kinase and RNase domains to generate functional outputs. The RNase activity of Ire1 plays a critical role in the UPR signaling pathway, where it can degrade large amounts of mRNA or process nonspecific mRNA to produce a transcriptional response (Hollien and Weissman, 2006). Previous studies have shown that the deletion of the Ire1 gene weakens the pathogenicity of *C. albicans* (Sircaik et al., 2021), suggesting that Ire1 plays a crucial role in regulating its pathogenicity. In summary, the highly conserved nature of the Ire1-dependent UPR signaling pathway indicates that it could serve as a foundation for the development of broad-spectrum antifungal drugs, with Ire1 representing a potential therapeutic target for *C. albicans*.

Our previous studies successfully established a *C. albicans* Ire1 gene knockout strain (designated Ire1*Δ*/Δ) and further investigated the role of the Ire1 gene in *C. albicans* ER stress (ERS) and pathogenic processes (Zhao et al., 2025). However, the effects of Ire1 small-molecule inhibitors on *C. albicans* and their potential therapeutic value have not been thoroughly explored. Therefore, in this study, we aim to investigate the feasibility of Ire1 RNase inhibitors in the pharmacological inhibition of *C. albicans*. We will further explore the impact of these inhibitors on pathogenic traits such as morphological transformation, adhesion ability, biofilm formation, and drug sensitivity. Additionally, we will examine their therapeutic potential in an animal model of intestinal colonization by *C. albicans*. This research may provide valuable pharmacological tools for future studies on how the *C. albicans* ER stress response pathway integrates with other signaling pathways to coordinate adaptive responses to various environmental stresses. Furthermore, it could offer new possibilities and foundational experimental data for optimizing treatment strategies for patients with *C. albicans* infections.

## 2 Materials and methods

### 2.1 Strains, growth conditions, and reagents

The pathogenic fungal strains used in this study included *C. albicans* SC5314, the Ire1*Δ*/Δ mutant strain, and its parental strain SN152. All strains were obtained from and preserved by the Biobank of the Department of Laboratory Medicine, Bethune International Peace Hospital (Hebei, China). Strains were routinely cultured on yeast peptone dextrose (YPD) solid medium composed of 1% (w/v) yeast extract, 2% (w/v) peptone, 2% (w/v) D-glucose, and 2% (w/v) agar (all from Oxoid Ltd., Basingstoke, United Kingdom) at 35°C. For liquid culture, a single colony was aseptically transferred into YPD broth (same formulation without agar) using a sterile inoculating loop. The cultures were incubated at 35°C with shaking at 160 rpm for 12–16 h to reach the logarithmic growth phase. Experimental reagents included 4μ8c, MKC8866, STF-083010, hygromycin B, carvacrol, itraconazole (ITZ), and fluconazole (FLU), all purchased from MedChemExpress (MCE, Monmouth Junction, NJ, United States). Tunicamycin (Tm) was obtained from Gibco (Thermo Fisher Scientific, Waltham, MA, United States). Antibiotics including streptomycin, ampicillin, gentamicin, and kanamycin were purchased from Solarbio Technology Co., Ltd. (Beijing, China). All reagents were prepared and stored according to the manufacturers’ instructions. Before use, they were diluted to working concentrations using sterile phosphate-buffered saline (PBS) or dimethyl sulfoxide (DMSO), depending on solubility requirements.

### 2.2 Molecular docking

The three-dimensional (3D) structures of the small-molecule compounds used in this study were retrieved from the PubChem database (National Center for Biotechnology Information, Bethesda, MD, United States).^1^ The amino acid sequence of the *C. albicans* SC5314 Ire1 protein was obtained from the NCBI protein database.^2^ Subsequently, the 3D structure of the Ire1 protein was predicted using AlphaFold 2.0 (DeepMind Technologies Limited, London, United Kingdom), and the resulting structure was downloaded in PDB format. Protein structure preprocessing and extraction of the RNase domain (amino acid residues 1,093–1,223) were performed using PyMOL 2.5 (Schrödinger, Inc., New York, NY, United States).^3^ Docking input files (PDBQT format) for the RNase domain and the small-molecule ligands were prepared using AutoDockTools 1.5.7 (The Scripps Research Institute, La Jolla, CA, United States). Molecular docking was then performed using AutoDock Vina 1.1.2 (The Scripps Research Institute, La Jolla, CA, United States) to predict the optimal binding conformations and calculate binding affinities. Finally, the docking results, including binding modes and hydrogen bond interactions, were visualized using PyMOL.

### 2.3 Real-time fluorescent quantitative polymerase chain reaction

Small-molecule compounds were added to YPD liquid medium, and *C. albicans* strains in the logarithmic growth phase were inoculated into the medium. After a certain period of incubation, cells were harvested for RNA extraction. Total RNA was extracted from both experimental and control groups using the Yeast RNA Extraction Kit (Mei5 Biotechnology Co., Ltd., Beijing, China). Complementary DNA (cDNA) was synthesized using the TransScript® First-Strand cDNA Synthesis SuperMix Kit (TransGen Biotech Co., Ltd., Beijing, China) according to the manufacturer’s instructions. Quantitative real-time PCR (qRT-PCR) was performed using 2 × HS SYBR Green qPCR Mix (Sanshi Biotech Co., Ltd., Shijiazhuang, Hebei, China) in an eight-well plate format. Each 25 μL reaction contained 1 μL of cDNA template, 0.4 μM of each forward and reverse primer, 12.5 μL of qPCR mix, and nuclease-free water. The thermal cycling conditions were as follows: initial denaturation at 95°C for 5 min, followed by 40 cycles of denaturation at 95°C for 5 s, annealing at 57°C for 15 s, and extension at 72°C for 30 s. The housekeeping gene ACT1 was used as the internal control to normalize gene expression levels. Relative gene expression was calculated using the 2^−ΔΔCt^ method (Vautier et al., 2015). Data are presented as the mean ± standard deviation (SD) from at least three biological replicates. The primer sequences used in this study are listed in Supplementary Table 1.

### 2.4 Agarose gel electrophoresis

After culturing *C. albicans* to the logarithmic growth phase, the target drug was added for treatment. Following a 2-h treatment, the yeast cells were collected, and total RNA was extracted from *C. albicans* using the same method as described above. cDNA was synthesized, and the cDNA was used as a template to amplify the Hac1u and Hac1s genes using the forward primer ATCATCAACCTCCCCTTCCT and the reverse primer TCAACATCATCTCCTAAAATCGAA. The amplification products were diluted three times and subjected to electrophoresis on a 4% agarose gel. The gel was then analyzed using a gel imaging system (Syngene, Cambridge, United Kingdom) for exposure and development, and the images were saved.

### 2.5 Growth curve measurement

For the growth curve analysis, *C. albicans* in the logarithmic growth phase was resuspended in fresh YPD liquid medium, and the yeast suspension was adjusted to a concentration of 2.0 × 10^6^ CFU/mL. The suspension was then treated with 4μ8c and incubated at 35°C in a shaking incubator at 160 rpm. Samples were taken at various time points to measure the optical density (OD) at 600 nm to assess the growth of the yeast.

### 2.6 Observation of solid plate colony morphology

An aliquot containing 2.5 × 10^3^ CFU of *C. albicans* in the logarithmic growth phase was spotted onto YPD agar plates or Spider agar plates containing or without 4μ8c. The Spider agar medium consists of 1% (w/v) mannitol (Solarbio Technology Co., Ltd., Beijing, China), 0.2% (w/v) K₂HPO₄ (Solarbio Technology Co., Ltd., Beijing, China), 1% (w/v) nutrient broth (Solarbio Technology Co., Ltd., Beijing, China), and 2% (w/v) agar. The plates were then incubated at 35°C in a static culture incubator for 5–10 days. Images were taken directly or observed and photographed using an inverted microscope.

### 2.7 Liquid hyphal induction experiment

*C. albicans* strains in the logarithmic growth phase were resuspended in RPMI 1640 liquid medium (Gibco, Thermo Fisher Scientific, Inc., Waltham, MA, United States) or Spider liquid medium, with or without the addition of 4μ8c. The initial culture concentration was adjusted to 2.0 × 10^6^ CFU/mL. Cultures were incubated at 35°C in a shaking incubator at 180 rpm. Samples were collected at 2, 4, 6, and 8 h, transferred to a 96-well plate, and observed under an inverted microscope to assess hyphal induction and morphological changes. Representative images were captured and recorded at each time point for comparative analysis.

### 2.8 Adhesion ability determination

*C. albicans* strains in the logarithmic growth phase were resuspended in RPMI 1640 liquid medium, either supplemented with or without 4μ8c, and adjusted to an initial concentration of 1.0 × 10^6^ CFU/mL. The suspensions were inoculated into sterile polystyrene 96-well plates, with three to five replicate wells per group. Plates were incubated at 35°C for 4 h to allow fungal adhesion. Following incubation, non-adherent cells were removed by washing the wells with sterile PBS. The remaining adherent cells were fixed with 100% methanol (Shanghai MacLean Biochemical Technology Co., Ltd., Shanghai, China) and stained with 0.5% (w/v) crystal violet (Solarbio Technology Co., Ltd., Beijing, China). Excess stain was removed, and the crystal violet bound to biofilms was solubilized using 33% (v/v) ice-cold acetic acid (McLean Biochemical Technology Co., Ltd., Shanghai, China) at 37°C. The absorbance was measured at 595 nm using a full-wavelength microplate reader, and results were recorded for quantitative analysis of adhesion capacity.

### 2.9 Determination of flocculation capacity

*C. albicans* strains in logarithmic growth phase were resuspended in RPMI 1640 or Spider liquid medium, either supplemented with or without 4μ8c. The initial concentration of each fungal suspension was adjusted to 5.0 × 10^6^ CFU/mL. Subsequently, 3.5 mL of each prepared suspension was transferred into separate glass test tubes and incubated at 35°C in a shaking incubator set at 180 rpm for 6–8 h. After incubation, the tubes were vortexed briefly for 5–10 s, followed by a resting period to allow settling. The degree of flocculation was visually assessed for each experimental group, and photographic documentation was obtained for record-keeping purposes.

### 2.10 Biofilm formation assay

*C. albicans* strains in the logarithmic growth phase were resuspended in RPMI 1640 liquid medium, either supplemented with or without 4μ8c. The cell suspension was adjusted to an initial concentration of 1.0 × 10^6^ CFU/mL. Subsequently, suspensions were inoculated into polystyrene 96-well plates with three to five replicate wells per experimental group and incubated at 35°C for 24 h. After incubation, wells were washed with PBS to remove non-adherent cells, followed by fixation with 100% methanol and staining with 0.5% (w/v) crystal violet. The bound dye was dissolved using 33% (v/v) acetic acid at 37°C, and OD_595_ was measured using a full-wavelength microplate reader.

### 2.11 Solid plate dilution assay

*C. albicans* cultures in logarithmic growth phase were adjusted to a concentration of 2.0 × 10^6^ CFU/mL and serially diluted in a 10-fold gradient, resulting in four dilutions per strain. Aliquots (2.5 μL) from each dilution were spotted onto YPD agar plates supplemented with or without the drug under investigation, beginning with the highest concentration. Plates were incubated at 35°C for 24–48 h, after which fungal growth was observed and photographically documented.

### 2.12 Establishment of intestinal infection model

Seven-week-old female BALB/c mice (18–20 g) were procured from Beijing Huafu Kang Biotechnology Co., Ltd. (Beijing, China) and randomly allocated into six experimental groups (*n* = 4 per group): Saline-1, SN152, Ire1*Δ*/Δ, Saline-2, 4μ8c, SC5314, and SC5314 + 4μ8c groups. To facilitate colonization, mice received drinking water containing streptomycin (2 mg/mL), penicillin (500 U/mL), gentamicin (0.1 mg/mL), and fluconazole (0.5 mg/mL) for 3 days. Fluconazole was subsequently withdrawn, while antibiotic treatment continued for an additional 24 h. Feces were collected post-antibiotic treatment, homogenized, and cultured on YPD agar plates (containing antibiotics), MacConkey agar, and blood agar plates to confirm microbiota depletion and dysbiosis (Supplementary Figure 1). Mice were then orally inoculated with 5 × 10^7^ CFU/animal of respective *C. albicans* suspensions (200 μL). Saline groups received physiological saline by oral gavage. Mice in the 4μ8c group were administered 4μ8c alone by oral gavage. The 4μ8c treatment group received 4μ8c (10 mg/kg) by oral gavage once daily, starting 8 h after SC5314 infection. Antibiotic administration continued in drinking water throughout the experiment. All procedures complied with the “National Regulations on the Administration of Animal Experiments” and were approved by the Animal Ethics Committee of Bethune International Peace Hospital (Approval No. 2024-KY-340).

### 2.13 Quantification of fungal load

Fecal and intestinal tissue samples, including luminal contents, were collected from infected mice at predetermined intervals, weighed, and homogenized at 40 mg/mL in sterile PBS using an ultrasonicator. Homogenates were plated onto YPD agar supplemented with ampicillin (100 μg/mL), kanamycin (50 μg/mL), and streptomycin (100 μg/mL), and incubated at 35°C for 24–48 h. Colony-forming units (CFU) were subsequently counted.

### 2.14 Histological analysis

Five days post-infection or post-treatment, mice were euthanized via cervical dislocation. Intestinal tissues were aseptically harvested, fixed in 4% paraformaldehyde solution (Biosharp, Shanghai, China) for 48 h, embedded in paraffin, sectioned (4 μm thickness), and stained with hematoxylin and eosin (HE) for histological examination.

### 2.15 Data analysis

All data are expressed as mean ± standard deviation (SD). Statistical analyses and graphical representations were conducted using GraphPad Prism 8.0 software (GraphPad Software, Inc., San Diego, CA, United States). Differences between two groups were evaluated using independent-samples *t*-tests, while differences among multiple groups were assessed using one-way analysis of variance (ANOVA) followed by Tukey’s multiple comparisons test. Statistical significance was defined as *p* < 0.05. The experiment was repeated at least three times.

## 3 Results

### 3.1 Molecular docking of Ire1 inhibitors with the Ire1 protein of *C. albicans*

Molecular docking methods (MDM) have increasingly become valuable tools in microbiology for exploring interactions between microbial protein targets and high-affinity ligands (Silva et al., 2019). AutoDock Vina, a widely utilized software for molecular docking and virtual screening (Trott and Olson, 2010), was employed in this study to evaluate the binding interactions between three small-molecule inhibitors (MKC8866, STF083010, and 4μ8c) and the Ire1 protein of *C. albicans*.

**Figure 1:**
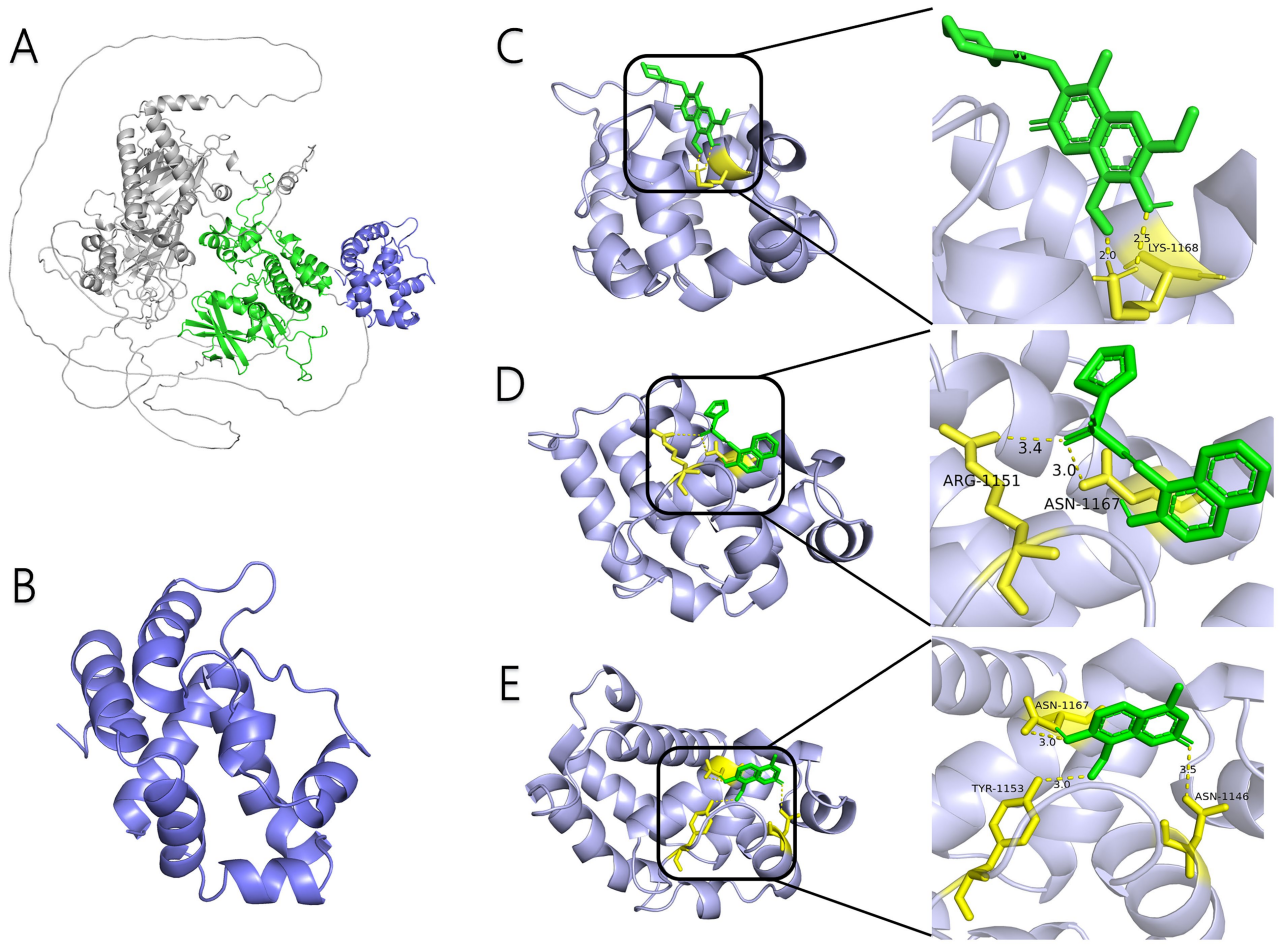
Interaction between small-molecule drugs and the Ire1 protein of *C. albicans*. **(A)** Predicted three-dimensional structure of the Ire1 protein from SC5314, generated using AlphaFold. The protein kinase domain is shown in green, and the RNase domain is shown in purple. **(B)** Three-dimensional structure of the Ire1 RNase domain, processed using PyMOL software. **(C)** Molecular docking visualization of MKC8866 binding to the Ire1 RNase domain. **(D)** Molecular docking visualization of STF038010 binding to the Ire1 RNase domain. **(E)** Molecular docking visualization of 4μ8c binding to the Ire1 RNase domain. In all panels, the ligand is shown in green, the receptor in purple, receptor amino acids interacting with the ligand are in yellow, and hydrogen bonds between the receptor and ligand are indicated by yellow dashed lines.

AlphaFold, a deep-learning-based predictive tool capable of accurately determining the three-dimensional structure of proteins, facilitates the understanding of protein functions and interactions, making it instrumental in drug target screening (Abramson et al., 2024; Jumper et al., 2021). In the present study, AlphaFold was first used to predict the three-dimensional structure of the Ire1 protein from the clinical standard strain SC5314 of *C. albicans* (Figure 1A). The amino acid residues from positions 769 to 1,090 correspond to the protein kinase domain, whereas residues 1,093 to 1,223 constitute the RNase domain (Table 1). Using PyMOL, extraneous amino acids were removed, isolating the RNase domain’s three-dimensional structure, which served as the receptor in docking analyses (Figure 1B). Subsequently, ligand structures for MKC8866, STF083010, and 4μ8c were retrieved from the PubChem database. Table 2 details the names and structures (both 2D and 3D) of these inhibitors. Docking simulations performed with AutoDock Vina predicted optimal binding sites and associated binding affinities of these inhibitors to the RNase domain. MKC8866 formed two hydrogen bonds with residue LYS-1168; STF083010 established hydrogen bonds with ARG-1151 and ASN-1167; and 4μ8c formed hydrogen bonds with ASN-1146, TYR-1153, and ASN-1167. Docking scores were −7.7 (MKC8866), −7.6 (STF083010), and −7.6 (4μ8c), indicating robust but modest binding affinities and suggesting potential inhibitory activity against the *C. albicans* Ire1 protein.

**Table 1:** Position of Ire1 major domain of *C. albicans.*

Description	Type	Position
Protein kinase	Domain	769–1,090
RNase	Domain	1,093–1,223

**Table 2:** List of ligands that bind to *C. albicans* Ire1 protein (both 2D and 3D structures were obtained from PubChem database).

Drug name	2D structure	3D structure	Docking score (kcal/mol)
MKC8866	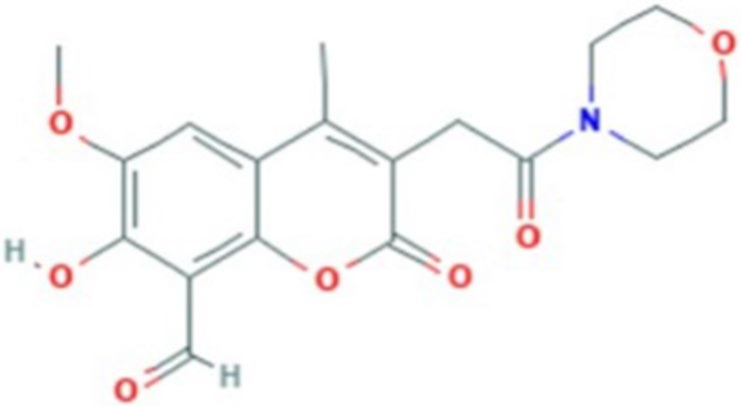	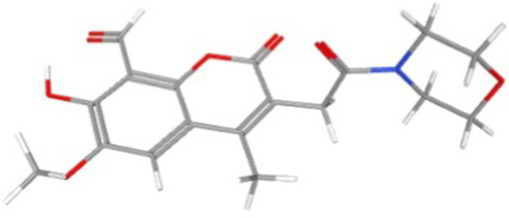	−7.7
STF083010	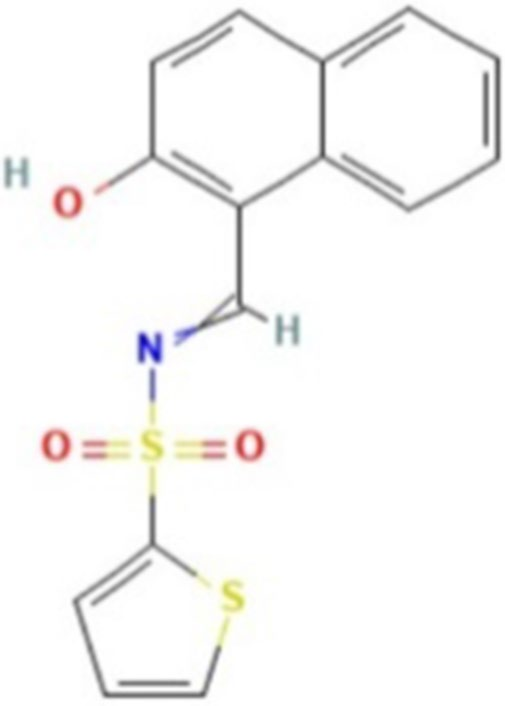	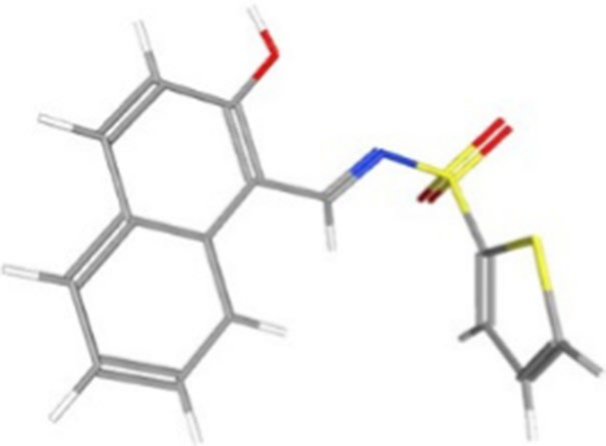	−7.6
4μ8c	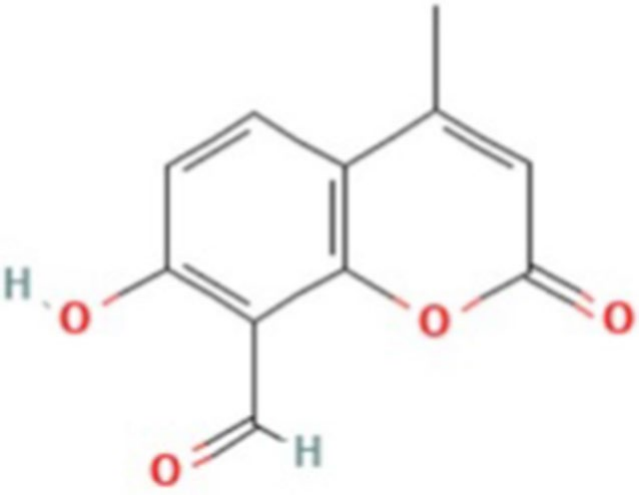	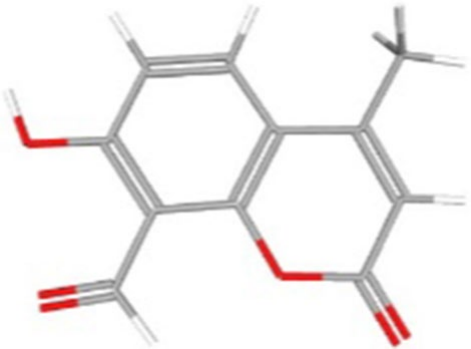	−7.6

### 3.2 4μ8c inhibits the expression of hac1s mRNA and UPR pathway target genes in *C. albicans*

Upon ERS, Ire1 dissociates from its chaperone Bip, leading to its oligomerization, autophosphorylation, and activation of its RNase domain. Activated Ire1 cleaves a 19-base pair noncanonical intron from Hac1u mRNA, generating spliced Hac1s mRNA. This spliced form translates effectively into the Hac1 protein, which regulates expression of UPR pathway target genes such as KAR2, SEC61, and YSY6, restoring ER homeostasis (Ishiwata-Kimata and Kimata, 2023; Wimalasena et al., 2008). Therefore, gene expression changes of Hac1s and related targets can serve as indicators for assessing Ire1 inhibitor efficacy.

**Figure 2:**
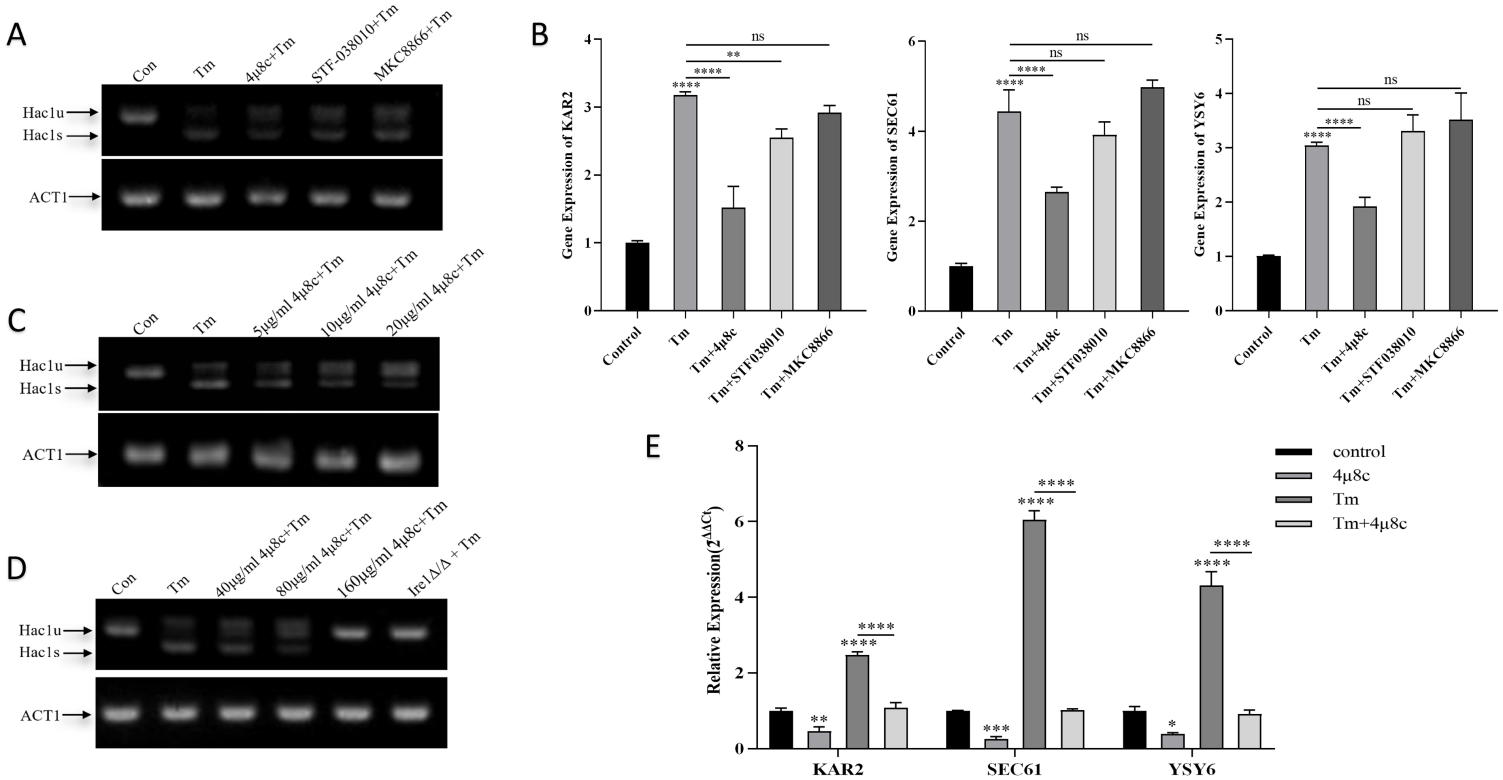
Analysis of Hac1 mRNA, KAR2, SEC61, and YSY6 expression in *C. albicans* following treatment with different drugs alone or in combination. Uncropped original gel images are provided in Supplementary Figures 3–5. **(A)** Qualitative analysis of Hac1 mRNA expression in *C. albicans* after treatment with 2 μg/mL Tm alone, three different Ire1 inhibitors (each at 40 μg/mL), and Tm in combination, as assessed by DNA electrophoresis. **(B)** Relative expression levels of the UPR pathway target genes KAR2, SEC61, and YSY6 in *C. albicans* following treatment with 2 μg/mL Tm alone, three different Ire1 inhibitors (each at 40 μg/mL), and Tm in combination. **(C,D)** Qualitative analysis of Hac1 mRNA expression in *C. albicans* following treatment with 2 μg/mL Tm, varying concentrations of 4μ8c alone, or in combination. **(E)** Relative expression levels of the UPR pathway target genes KAR2, SEC61, and YSY6 in *C. albicans* after treatment with 2 μg/mL Tm, 160 μg/mL 4μ8c, alone or in combination (Tm and the inhibitor were administered simultaneously during co-treatment) (ns *p* ≥ 0.05, *p < 0.05, **p < 0.01, ***p < 0.001, ****p < 0.0001).

This study employed RT-qPCR and agarose gel electrophoresis to analysis gene expression in *C. albicans* exposed to three inhibitors (40 μg/mL each) alongside tunicamycin (Tm), a potent inducer of ERS through inhibition of N-linked glycosylation (Guillemette et al., 2011). Compared with the control group, treatment with tunicamycin (Tm) led to increased expression of Hac1s and UPR target genes in *C. albicans* (Figure 2). Notably, co-treatment with 4μ8c and Tm significantly reduced the expression levels of Hac1s, as well as the UPR-related genes KAR2, SEC61, and YSY6, suggesting that 4μ8c effectively inhibited activation of the UPR pathway under acute ER stress conditions (Figures 2A,B). By contrast, MKC8866 and STF083010 did not exhibit clear inhibitory effects under the conditions applied in this study. Concentration optimization studies revealed that 160 μg/mL of 4μ8c completely suppressed Hac1s mRNA, mirroring results obtained with the Ire1*Δ*/Δ strain, and significantly downregulated UPR pathway targets, defining this concentration for subsequent experiments (Figures 2C–E).

### 3.3 4μ8c impairs morphological transformation of *C. albicans*

**Figure 3:**
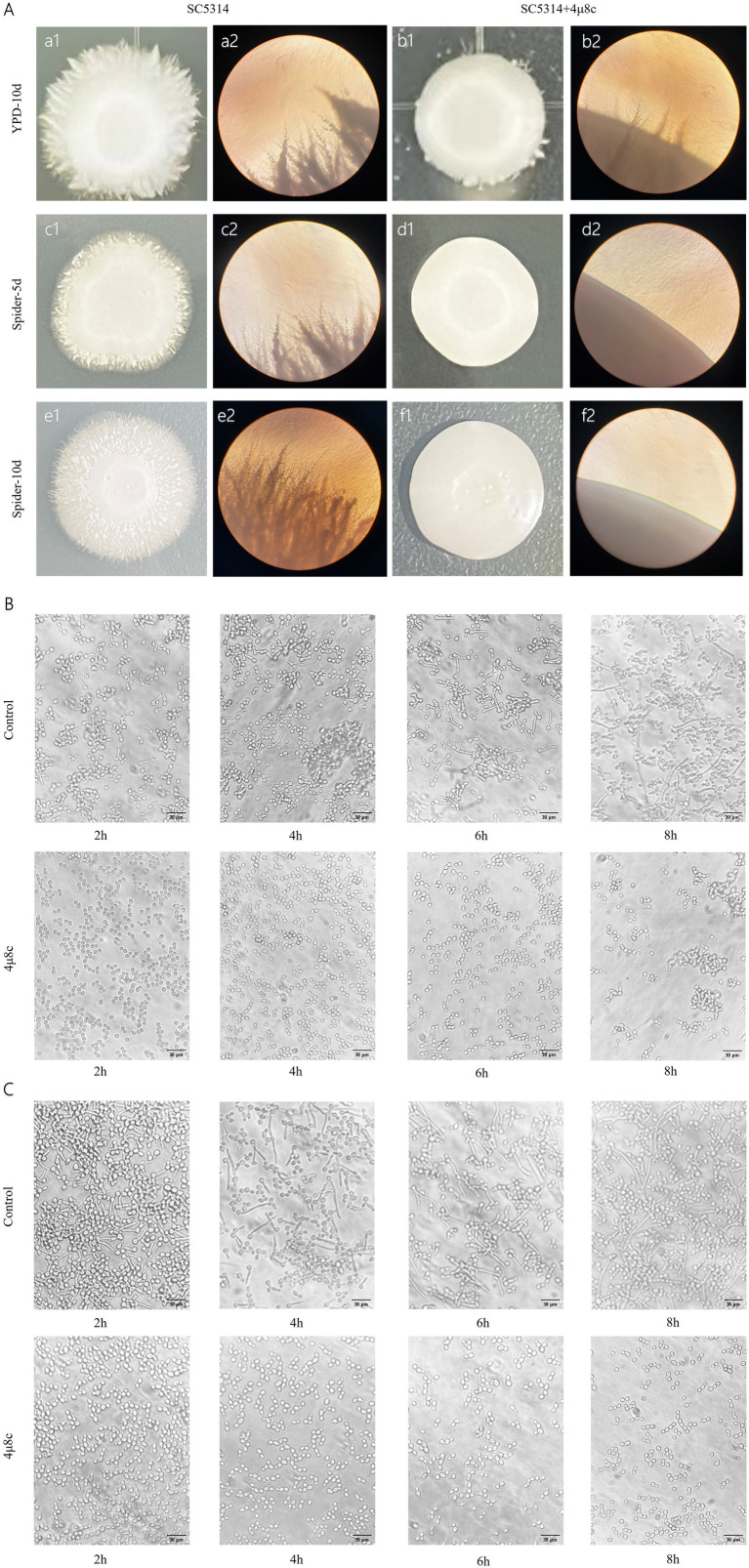
Effect of 4μ8c on the morphological transformation of *C. albicans*. **(A)** Inhibitory effect of 4μ8c on the morphological transformation of *C. albicans* colonies grown on solid medium (100×). **(B)** Inhibitory effect of 4μ8c on the dynamic formation of hyphae in *C. albicans* in RPMI 1640 + 10% FBS liquid medium (400×). **(C)** Inhibitory effect of 4μ8c on the dynamic formation of hyphae in *C. albicans* in Spider liquid medium (400×).

Morphological transformation is critical for *C. albicans* pathogenicity, aiding host tissue invasion and adaptation to environmental stresses (Chen et al., 2020; Lopes and Lionakis, 2022). Solid plate assays and liquid medium induction studies examined the effect of 4μ8c on morphological conversion. On YPD and Spider agar plates without 4μ8c, *C. albicans* colonies exhibited irregular edges, while the addition of 4μ8c resulted in smoother colony surfaces (Figure 3A). Observations under an inverted microscope were consistent with the results seen on the agar plates (Figure 3A). Microscopic evaluation of liquid cultures confirmed reduced hyphal growth in 4μ8c-treated groups compared to untreated controls, indicating significant impairment of morphological conversion by 4μ8c (Figures 3B,C).

### 3.4 4μ8c impairs the adhesion and flocculation abilities of *C. albicans*

**Figure 4:**
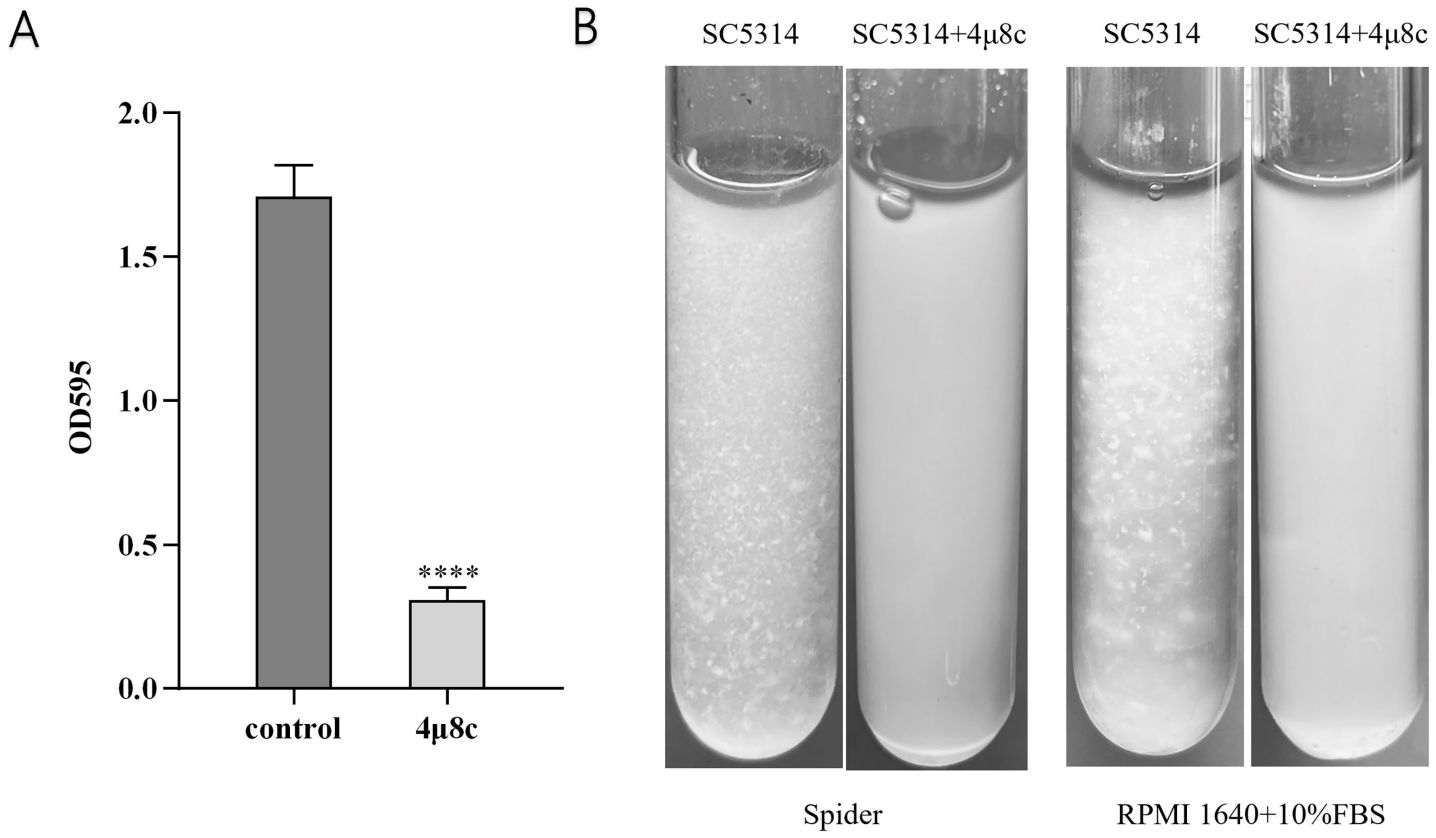
Effect of 4μ8c on the in vitro adhesion and flocculation abilities of *C. albicans*. **(A)** Inhibitory effect of 4μ8c on the adhesion ability of *C. albicans* assessed using a polystyrene microplate model (****p < 0.0001). **(B)** Inhibitory effect of 4μ8c on the flocculation ability of *C. albicans* studied in Spider and RPMI 1640 + 10% FBS liquid culture media.

Adhesion and flocculation abilities are integral to *C. albicans* colonization and pathogenicity (Jung et al., 2020; Wei et al., 2023). To assess these properties, we performed adhesion and flocculation assays on the strains. In the adhesion assay, *C. albicans* cells were incubated in polystyrene plates with or without 4μ8c, followed by crystal violet staining and quantitative analysis. Treatment with 4μ8c significantly reduced adhesion to the polystyrene surface by 81.96% (*p* < 0.0001) (Figure 4A). In addition, RPMI 1640 medium supplemented with 10% FBS and Spider medium were used as hyphal inducers to assess the effect of 4μ8c on flocculation after a defined induction period. The results showed that in the control group, *C. albicans* exhibited prominent clumping, while cultures treated with 4μ8c appeared uniformly turbid, indicating a significant suppression of flocculation (Figure 4B).

### 3.5 4μ8c attenuates biofilm formation by *C. albicans*

**Figure 5:**
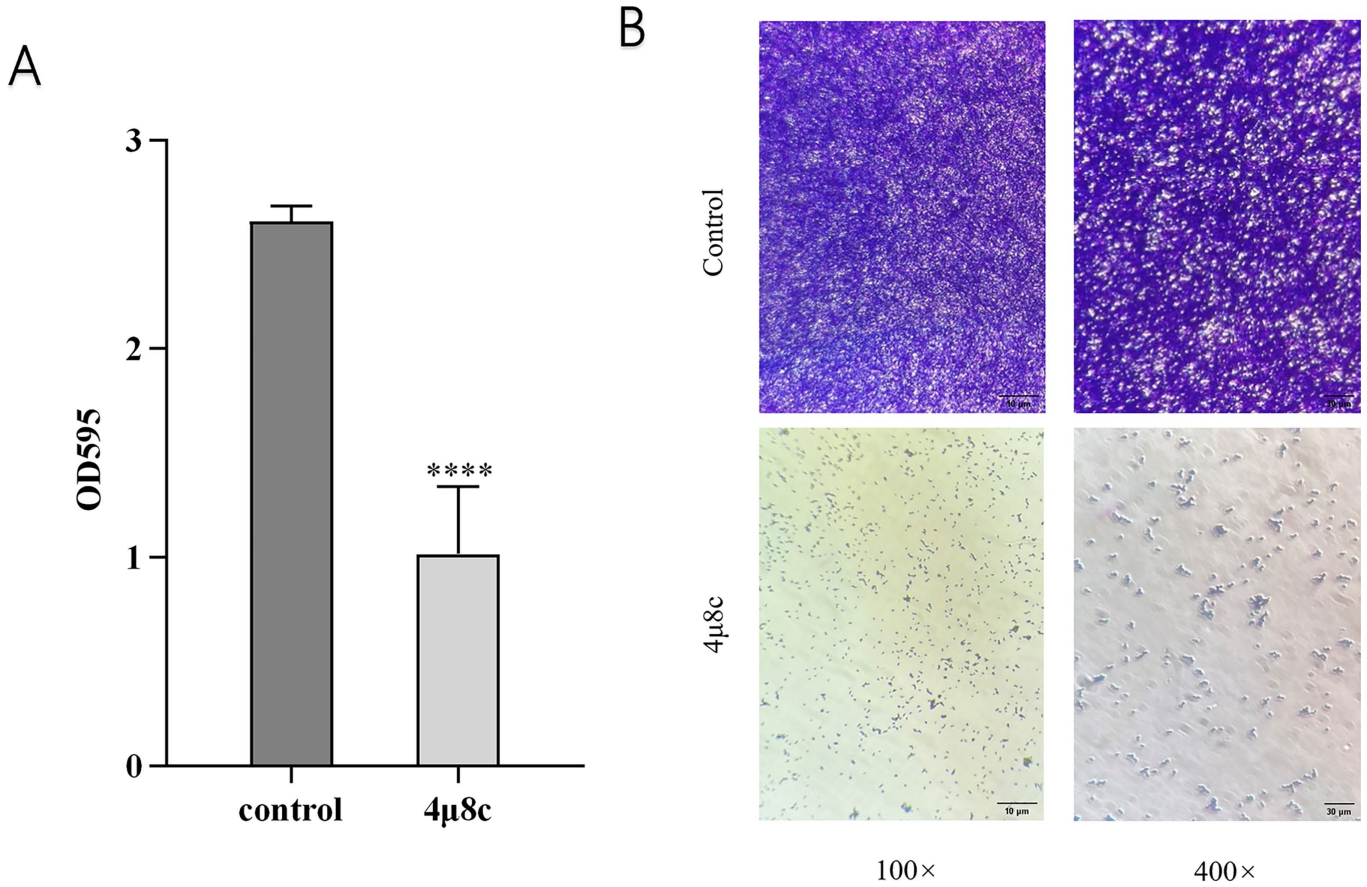
Effect of 4μ8c on the in vitro biofilm formation ability of *C. albicans*. **(A)** Inhibitory effect of 4μ8c on the biofilm biomass of *C. albicans* in vitro (****p < 0.0001). **(B)** Direct visualization of the inhibitory effect of 4μ8c on *C. albicans* biofilm formation in vitro using an inverted microscope.

Crystal violet staining is commonly employed for quantifying biofilm biomass (Meliefste et al., 2024). In this study, we utilized crystal violet staining to assess the impact of 4μ8c on biofilm formation by *C. albicans*. Results demonstrated in Figure 5A reveal that the biofilm biomass in the 4μ8c-treated group was significantly reduced by 61.07% compared to the control group (*p* < 0.0001), indicating a robust inhibitory effect of 4μ8c on *C. albicans* biofilm formation *in vitro*. Moreover, inverted microscopic observation further corroborated these findings. While the control group displayed dense, mature, and multilayered biofilms, the 4μ8c-treated group primarily exhibited isolated yeast-form cells, clearly indicating significant biofilm disruption induced by 4μ8c (Figure 5B).

### 3.6 Effect of 4μ8c on antifungal sensitivity of *C. albicans*

**Figure 6:**
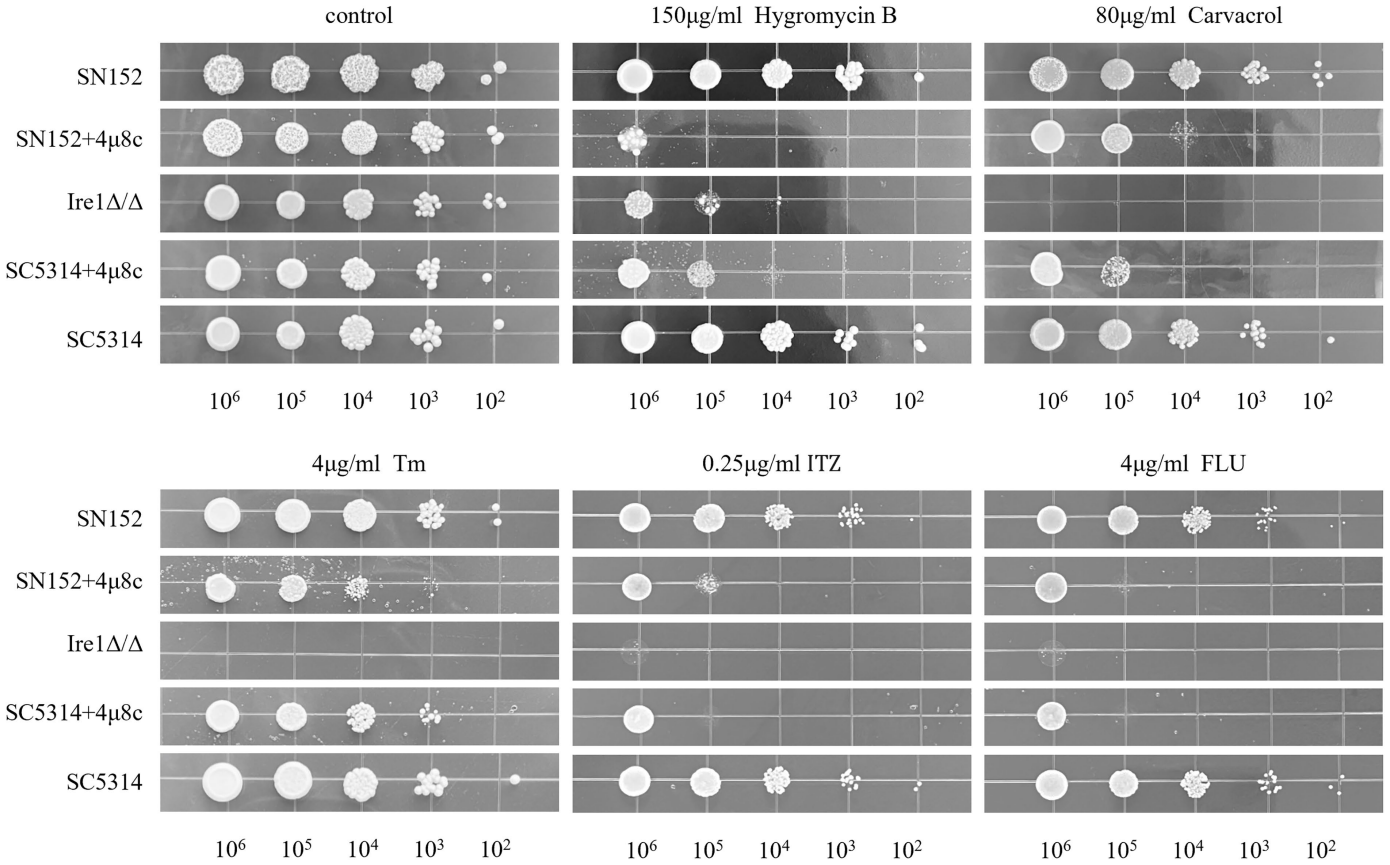
Solid plate pressure-dependent phenotype assay. *C. albicans* cultures (SC5314, Ire1*Δ*/Δ strain, and the parental strain SN152 of the Ire1Δ/Δ strain), with an initial concentration of 10^6^ CFU/mL, along with their serial 10-fold dilutions, were spotted from left to right on YPD solid plates containing hygromycin B, carvacrol, Tm, ITZ, FLU in the presence or absence of 4μ8c and incubated for 48 h to observe growth. Uncropped original growth images are provided in Supplementary Figure 6.

We investigated the influence of 4μ8c on the antifungal susceptibility of the standard *C. albicans* strain SC5314, the Ire1*Δ*/Δ mutant strain, and its parental strain SN152, against various antifungal agents, including hygromycin B, carvacrol, Tm, ITZ, and FLU. As depicted in Figure 6, the Ire1Δ/Δ strain exhibited markedly increased sensitivity to these antifungal agents relative to the SN152 parental strain. Although 4μ8c alone minimally impacted fungal growth, co-treatment with 4μ8c significantly augmented the inhibitory effects of the antifungal drugs tested on SC5314 and SN152 strains. Thus, 4μ8c enhances the susceptibility of *C. albicans* to Hygromycin B, carvacrol, Tm, ITZ, and FLU.

### 3.7 4μ8c suppresses expression of pathogenicity-associated genes in *C. albicans*

**Figure 7:**
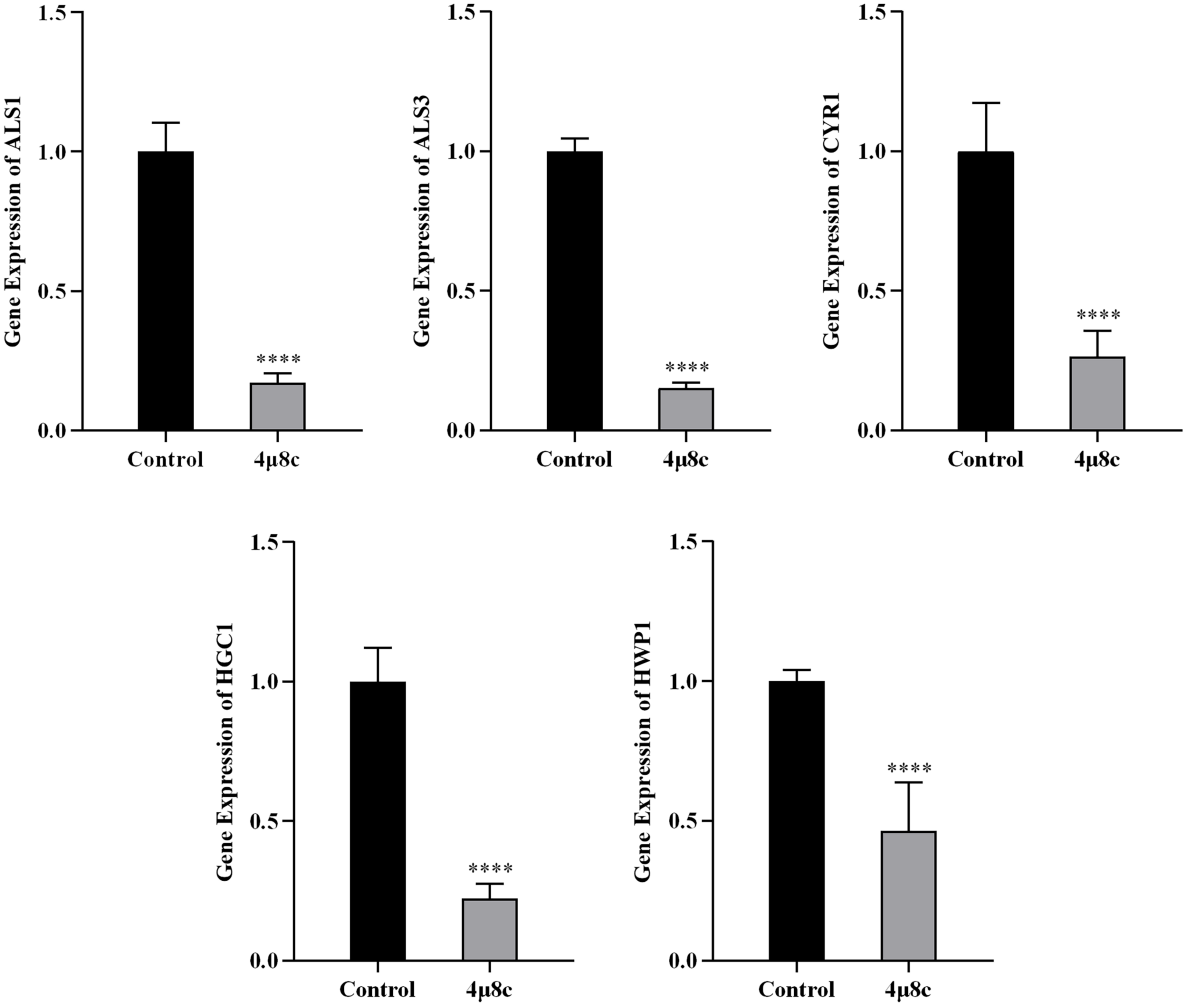
4μ8c inhibits the expression of genes associated with the pathogenicity of *C. albicans*. The relative expression levels of the target genes were measured by RT-qPCR analysis and calculated using the 2^-∆∆CT^ method (****p < 0.0001).

We assessed the impact of 4μ8c on pathogenicity-related gene expression in the clinical standard strain SC5314 by RT-qPCR (Figure 7). The analyzed genes, including *ALS1*, *ALS3*, *CYR1*, *HGC1*, and *HWP1*, are critical to adhesion, hyphal growth, and biofilm formation. Treatment with 4μ8c significantly downregulated expression levels of all evaluated genes compared to controls (*p* < 0.0001), highlighting the compound’s potential to reduce the virulence traits of *C. albicans*.

### 3.8 Impact of Ire1 gene deletion on fungal colonization in a mouse intestinal model

**Figure 8:**
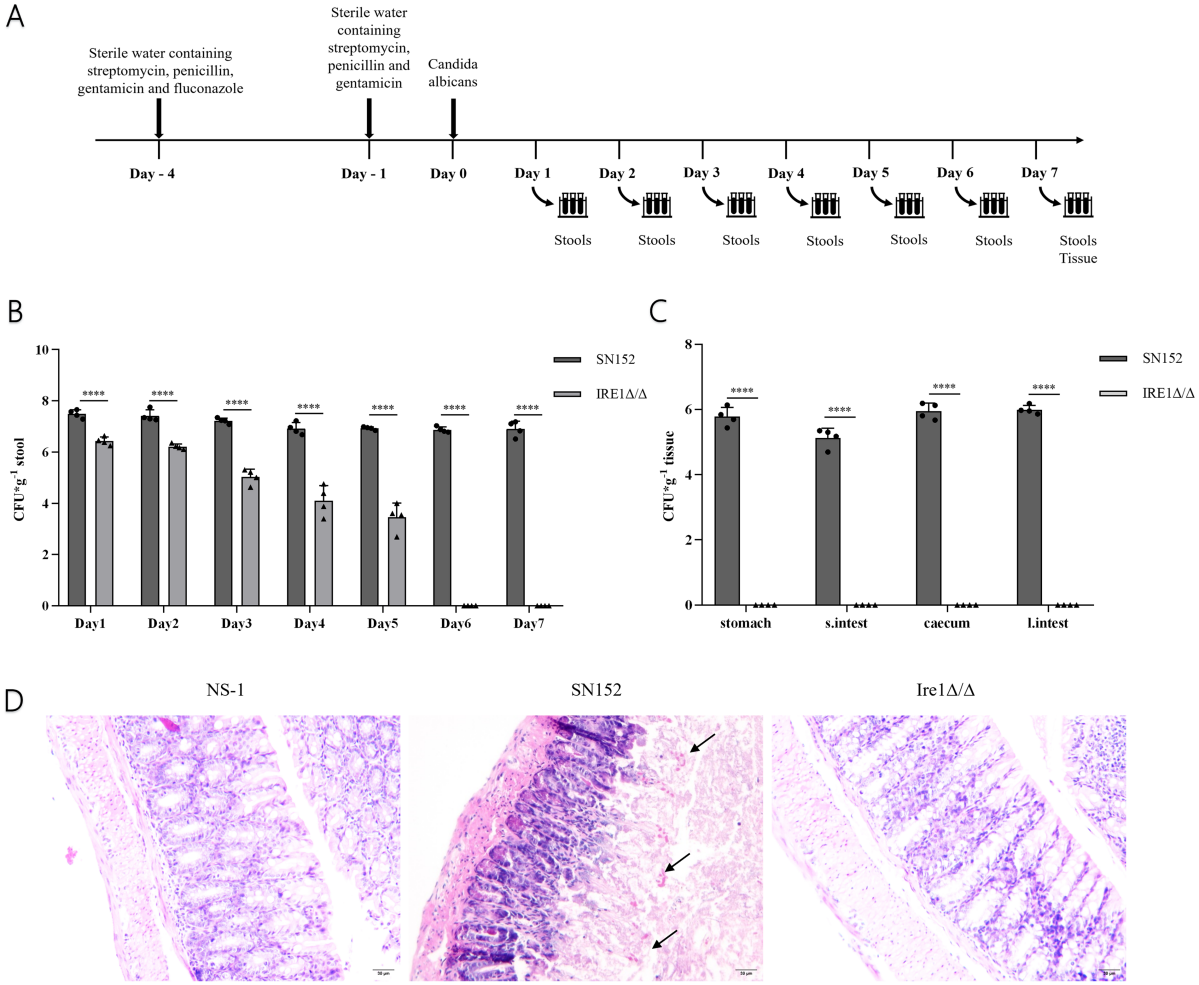
Fungal load and pathological analysis of feces, intestinal tissues, and contents in the SN152 and Ire1Δ/Δ intestinal colonization mouse model. **(A)** Flowchart of the *C. albicans* intestinal infection and colonization model. **(B)** Fungal load in the feces of intestinal colonization model mice from days 1 to 7 post-infection. **(C)** Fungal load in gastrointestinal tissues obtained from intestinal colonization model mice after decapitation on day 7 of infection. **(D)** HE staining of intestinal mucosal sections from intestinal colonization model mice collected after decapitation on day 7 of infection (ns *p* ≥ 0.05, *p < 0.05, **p < 0.01, ***p < 0.001, ****p < 0.0001).

We utilized a mouse intestinal colonization model to evaluate the role of the Ire1 gene and associated pharmacological interventions in the colonization and pathogenicity of C. albicans (Figure 8A), based on established methodologies (Koh, 2013; Koh et al., 2008; Vautier et al., 2015). Mice received a sterile antibiotic solution containing streptomycin, penicillin, and gentamicin to disrupt gut microbiota. Post-antibiotic treatment significantly reduced gut microbiota levels (Supplementary Figure 2), enabling controlled colonization experiments. Fungal load assessments demonstrated that intestinal colonization by the Ire1Δ/Δ strain significantly decreased shortly after infection initiation and fell below detectable limits by day 6. Compared to SN152-infected controls, the fungal load was significantly lower at all measured time points (days 1–7; P < 0.05) (Figure 8B) (Supplementary Table 2). Additionally, fungal burdens in gastrointestinal tissues were also significantly diminished (Figure 8C) (Supplementary Table 3), with histological examination revealing reduced intestinal damage in the Ire1Δ/Δ-infected group compared to pronounced mucosal injury observed in SN152 controls (Figure 8D). These data strongly suggest that the Ire1 gene is essential for effective intestinal colonization by C. albicans.

### 3.9 Influence of 4μ8c on fungal colonization in a mouse intestinal model

**Figure 9:**
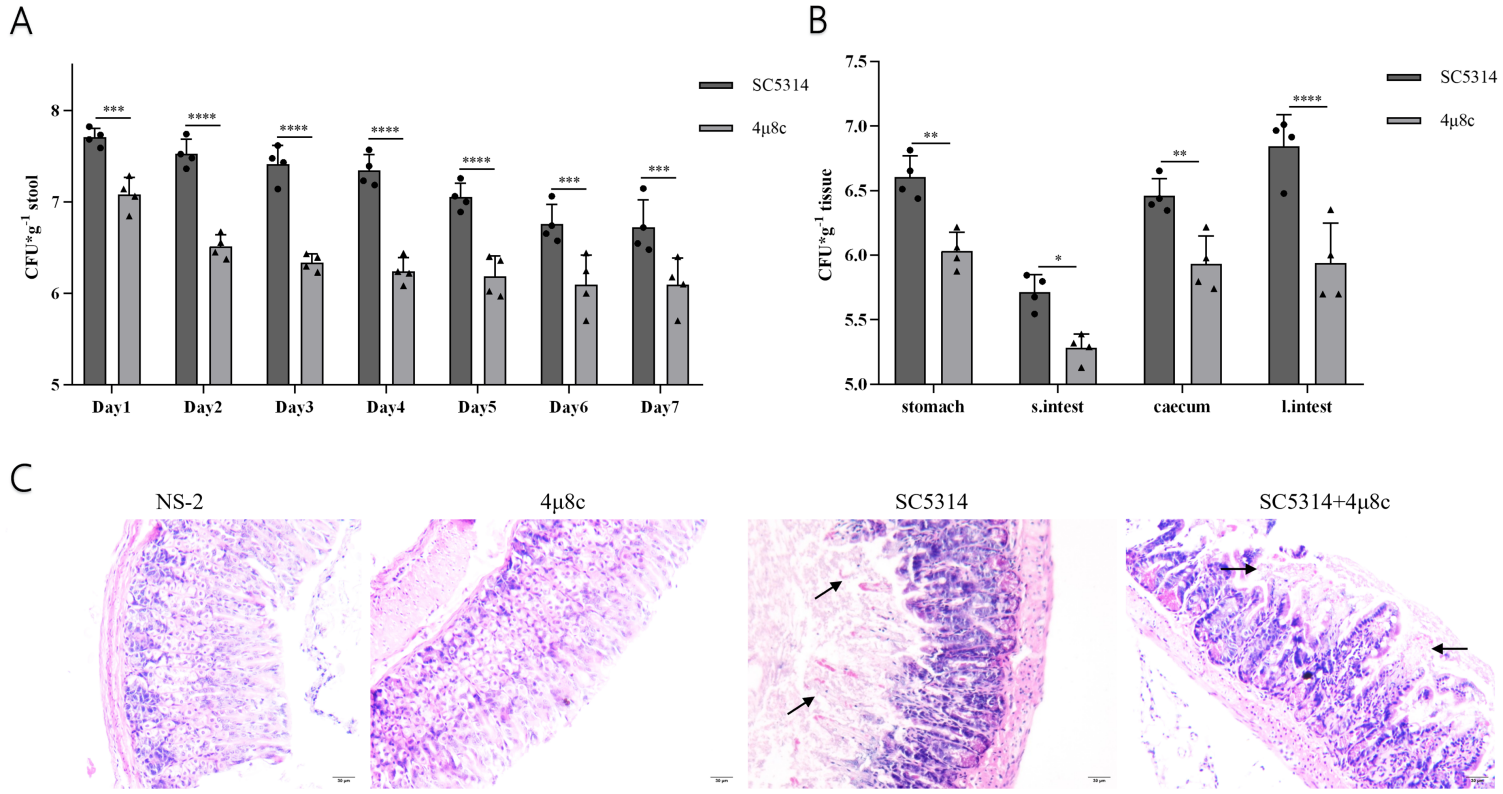
Fungal load and pathological analysis of feces, intestinal tissues, and contents in the SN152 and 4μ8c-Treated Intestinal colonization mouse model. **(A)** Fungal load in the feces of intestinal colonization model mice infected from days 1 to 7 post-infection. **(B)** Fungal load in gastrointestinal tissues obtained from intestinal colonization model mice euthanized by decapitation on day 7 of infection. **(C)** HE staining of intestinal mucosal sections from intestinal colonization model mice collected after decapitation on day 7 post-infection. (ns *p* ≥ 0.05, *p < 0.05, **p < 0.01, ***p < 0.001, ****p < 0.0001).

We further investigated the therapeutic potential of 4μ8c on intestinal colonization by *C. albicans*. Fungal load measurements revealed significantly reduced fecal colonization in 4μ8c-treated mice at all assessed time points (days 1–7; *p* < 0.001) compared to untreated SC5314 controls (Figure 9A; Supplementary Table 4). Similar significant reductions were observed in fungal loads within various gastrointestinal regions (stomach, small intestine, cecum, large intestine) and their contents (*p* < 0.05) (Figure 9B) (Supplementary Table 5). Histological analyses confirmed substantially mitigated intestinal mucosal damage in 4μ8c-treated mice, characterized by reduced villous disruption and preserved epithelial integrity (Figure 9C). These findings collectively demonstrate that 4μ8c effectively reduces intestinal colonization and tissue damage caused by *C. albicans*, underscoring its promising therapeutic potential.

## 4 Discussion

*C. albicans* is an opportunistic fungal pathogen capable of causing a range of diseases, from mucosal infections to invasive infections (Kumamoto et al., 2020; Romo and Kumamoto, 2020). Its adaptability and pathogenicity depend on the proper functioning of the ER. Under physiological conditions, protein folding demands are tightly coordinated with ER capacity. However, under adverse environmental stresses such as oxidative insult or nutrient deprivation, unfolded or misfolded proteins accumulate and form toxic aggregates, thereby disrupting ER homeostasis and threatening cellular viability (Deshpande et al., 2008; De Pereira Sa and Del Poeta, 2022). As toxic proteins accumulate, cells activate the UPR signaling pathway, initiating a series of adaptive responses to maintain ER homeostasis. These responses include reducing the influx of newly synthesized proteins into the ER, enhancing protein transport from the ER, increasing the expression of ER-associated molecular chaperones and folding enzymes, and promoting the degradation of unfolded or misfolded proteins (Guirao-Abad et al., 2022; Malhotra and Kaufman, 2007). Thus, the maintenance of ER function is essential for *C. albicans* survival, stress tolerance, and virulence within the host.

In this study, we first employed molecular docking modeling (MDM) to evaluate the binding affinities of three small-molecule inhibitors to *C. albicans* Ire1, the primary ER stress sensor in fungi. All three compounds demonstrated effective binding, with affinities of −7.7, −7.6, and −7.6 kcal/mol, respectively (Table 2). Based on these findings, we hypothesized that these compounds may inhibit Ire1 activity and thereby attenuate UPR signaling. Tm was utilized to pharmacologically induce ERS. Results showed that 4μ8c significantly suppressed UPR activation in *C. albicans* under ERS conditions (Figures 2A,B). Dose–response analyses revealed that 160 μg/mL of 4μ8c yielded the most effective inhibition and was subsequently used in downstream experiments (Figures 2C–E).

The morphological transition, adhesion, flocculation, and biofilm formation abilities of *C. albicans* are crucial for its pathogenicity. *C. albicans* can switch freely between yeast, pseudohyphal, and hyphal forms, and this ability to undergo morphological transitions is a key mechanism for its invasion and spread (Lindemann-Perez and Perez, 2024; Prasad and Tippana, 2023). Adhesion is the initial step in *C. albicans* infection, allowing it to adhere to host tissues or artificial materials such as endotracheal tubes, providing a foundation for subsequent infection (Jung et al., 2020; Wei et al., 2023). Flocculation reflects interactions between *C. albicans*, facilitating the formation of its collective behavior. Additionally, *C. albicans* can form biofilms, which are highly resistant to antifungal treatments and host immune responses (Wall et al., 2019). Prior studies have shown that Ire1 is the sole ER stress receptor in fungi, and its deletion alters fungal virulence traits (Sircaik et al., 2021; Zhao et al., 2025). To further explore whether 4μ8c mimics the phenotypes of Ire1-deficient strains, we assessed its effects on several pathogenic features of *C. albicans*. Notably, 4μ8c had no impact on fungal proliferation under standard conditions (Supplementary Figure 2). However, it significantly impaired key virulence-associated traits, including hyphal morphogenesis, adhesion, flocculation, and biofilm formation, as demonstrated through solid media growth assays, hyphal induction assays (Figure 3), and adhesion, flocculation, and biofilm quantification tests (Figures 4, 5).

To investigate potential alterations in antifungal susceptibility, we assessed the effects of 4μ8c on *C. albicans* resistance to Hygromycin B, carvacrol, Tm, ITZ, and FLU using spot dilution assays. Hygromycin B is an aminoglycoside antibiotic purified from *Streptomyces hygroscopicus*. It primarily inhibits normal protein synthesis by interfering with 70S ribosome translocation and inducing misreading of the mRNA template, leading to cell death in bacteria, fungi, and mammalian cells (Moreno-Martinez et al., 2015; Vallieres et al., 2018). Carvacrol, a component of aromatic plants such as thyme and oregano, perturbs ER membrane integrity and induces ER stress (Chaillot et al., 2015). Tm directly induces ER stress (Yang et al., 2021), while ITZ and FLU, both azole antifungals, inhibit ergosterol biosynthesis and compromise fungal membrane integrity (Pristov and Ghannoum, 2019). Our results showed that both Ire1*Δ*/Δ strains and 4μ8c-treated *C. albicans* exhibited increased sensitivity to FLU, Tm, and carvacrol (Figure 6), suggesting that Ire1 is essential for stress adaptation and drug resistance. This effect is likely mediated through suppression of UPR signaling, leading to reduced environmental resilience and increased antifungal susceptibility.

To further elucidate the molecular mechanisms underlying the observed phenotypic changes, we quantified the expression levels of key pathogenicity-associated genes—*ALS1*, *ALS3*, *CYR1*, *HGC1*, and *HWP1*—using RT-qPCR. *ALS1* and *ALS3* play key roles in the adhesion, invasion, and biofilm formation of *C. albicans*, directly influencing its pathogenicity and ability to adapt to the host (Hoyer and Cota, 2016). *CYR1* is a critical component of the Ras/cAMP/PKA signaling pathway, which plays a pivotal role in regulating *C. albicans* pathogenicity. It is essential for the regulation of hyphal formation, adhesion, invasion, and responses to various stressors such as temperature and nutrient deprivation (Huang et al., 2019). *HWP1* encodes a cell wall mannoprotein that is important for hyphal growth and acts as a significant adhesion molecule involved in developmental regulation, with both antigenic and hyphal specificity (Fan et al., 2013). *HGC1* is a hyphal-specific gene, and deletion of *HGC1* leads to defects in hyphal growth. The expression of *HGC1* is regulated by the cAMP/PKA signaling pathway and the transcriptional repressors Tup1/Nrg1, in coordination with other virulence genes like *HWP1* (Zheng et al., 2004). These genes collectively enhance the adaptability and pathogenicity of *C. albicans* within the host. 4μ8c treatment significantly downregulated the expression of all five genes (Figure 7), correlating with the attenuated pathogenic traits observed *in vitro*. This also suggests that the reduced pathogenicity of *C. albicans* may not be solely mediated by the Ire1-dependent UPR pathway, but may involve interactions with other signaling pathways.

Given that intestinal dysbiosis promotes *C. albicans* overgrowth and translocation (Koh, 2013; Proctor et al., 2023), we established a murine model of intestinal colonization by disrupting the gut microbiota with a combination of streptomycin, penicillin, and gentamicin (Figure 8A). In this study, the Ire1*Δ*/Δ strain showed a significant reduction in its colonization ability in the mouse intestine, with fungal load dropping below the limit of detection by day 6 (Figures 8B,C), and histopathological analysis revealed no significant damage (Figure 8D), suggesting that Ire1 plays a crucial role in the colonization and pathogenicity of *C. albicans*. Ire1 is a central factor in the endoplasmic reticulum stress response (ERS) pathway and is involved in regulating fungal adaptation to external stressors (Hernandez-Elvira et al., 2018). In the absence of Ire1, *C. albicans* may fail to effectively colonize under various environmental stresses, such as oxidative stress in the host, highlighting the importance of ER stress response in fungal infections. Previous studies have demonstrated that the pathogenicity of the Ire1Δ/Δ strain, including its morphological transition, adhesion, and biofilm formation, is significantly reduced, and that it is avirulent in invasive *Candida* infections, with no effect on the survival rate of infected mice (Zhao et al., 2025). The results of this study further suggest that Ire1 not only plays an important role in the *in vitro* pathogenicity and invasive infections of *C. albicans*, but also significantly affects its colonization within the host intestine. Previous studies have reported the safety of 4μ8c. Mimura et al. (2012) administered 4μ8c (10 mg/kg) in a multiple myeloma mouse xenograft model and observed effective tumor suppression without systemic toxicity or weight loss. Tufanli et al. (2017) further confirmed the safety of intraperitoneal injection of 4μ8c (20 mg/kg) in ApoE−/− mice, with no observable adverse effects. Kamath et al. (2024) applied 4μ8c topically in a murine fungal keratitis model at concentrations up to 2.5 mM (510.45 μg/mL) without inducing tissue inflammation or structural damage. These results support the safety of the drug at the doses used in this study. In our study, a dosage of 10 mg/kg was selected as an exploratory *in vivo* dose, and no apparent toxicity—such as weight loss or behavioral abnormalities—was observed in the treated mice during the course of treatment. The results of the study revealed mice treated with 4μ8c showed a downward trend in *C. albicans* fungal load in the intestine (Figures 9A,B). Histopathological analysis revealed that intestinal mucosal damage remained present in the treatment group, but was less severe compared to the untreated group (Figure 9C). This suggests that 4μ8c plays a beneficial role in inhibiting *C. albicans* colonization, although its effect on repairing infection-induced intestinal damage appears limited. This phenomenon may be closely linked to chronic immune responses and the restoration of intestinal barrier function. These findings suggest that while 4μ8c, as an Ire1 inhibitor, significantly affects the pathogenicity of *C. albicans* in vitro, more comprehensive therapeutic effects could be achieved by combining it with other strategies, particularly those targeting the restoration of intestinal barrier function.

Collectively, our findings suggest that Ire1 plays a central role in regulating *C. albicans* virulence, stress adaptation, and intestinal colonization. Pharmacological inhibition of Ire1 using 4μ8c represents a promising antifungal strategy. However, the specific effects of 4μ8c on host immune responses, as well as the potential crosstalk between the UPR pathway and other signaling cascades involved in *C. albicans* pathogenicity, remain to be fully elucidated. In addition, further investigations are needed to assess host cell tolerance at the selected dose, along with dose–response and pharmacokinetic analyses, to better define the therapeutic potential of 4μ8c in vivo. Although no apparent toxic manifestations were observed in treated animals, a comprehensive toxicological evaluation, including cytotoxicity assays, pharmacokinetic analysis, and long-term safety assessment, will be essential in future investigations to determine the therapeutic window of 4μ8c and to support its potential clinical application. In addition, exploring the potential in vivo synergistic effects of 4μ8c in combination with antifungal agents such as ITZ or FLU, as well as investigating additional small-molecule inhibitors targeting Ire1 in *C. albicans*, may also provide valuable insights for future therapeutic strategies.

## 5 Conclusion

4μ8c inhibits the function of *C. albicans* Ire1 and reduces the expression of UPR pathway target genes under ER stress conditions. By inhibiting Ire1, 4μ8c attenuates *C. albicans* morphological transition, hyphal formation, adhesion, flocculation, and biofilm formation abilities, while also enhancing its sensitivity to various drugs, including hygromycin B, carvacrol, Tm, ITZ, and FLU. Furthermore, *C. albicans* Ire1 plays a critical role in intestinal colonization, and 4μ8c shows potential therapeutic effects in the intestinal colonization infection model.

## Data Availability

The raw data supporting the conclusions of this article will be made available by the authors, without undue reservation.

## Footnotes

^1^ https://pubchem.ncbi.nlm.nih.gov/

^2^ https://www.ncbi.nlm.nih.gov/

^3^ https://pymol.org/
