# Correction: Ire1 inhibitors attenuate *Candida albicans* pathogenicity and demonstrate potential for application in antifungal therapy

**DOI:** 10.3389/fmicb.2025.1721908

**Published:** 2025-10-23

**Authors:** Hua Wang, Mengyan Li, Qiuyue Wang, Huihai Zhao, Mengyu Jiang, Qi Cui, Daxin Lei, Keran Jia, Fukun Wang

**Affiliations:** Department of Clinical Laboratory, Bethune International Peace Hospital, Shijiazhuang, China

**Keywords:** *Candida albicans*, endoplasmic reticulum, Ire1, Ire1 inhibitor, 4μ8c, pathogenicity

Affiliation “Department of Clinical Laboratory, Bethune International Peace Hospital, Shijiazhuang, China” was omitted for author Mengyan Li

This affiliation has now been added for author Mengyan Li.

Author Mengyan Li was erroneously assigned to affiliation Department of Clinical Laboratory, The First Hospital of Hebei Medical University, Shijiazhuang, China.

This affiliation has now been removed for author Mengyan Li.

The original version of this article has been updated.

