# Supplementary material for: Ire1 inhibitors attenuate Candida albicans pathogenicity and demonstrate potential for application in antifungal therapy
Source: Front Microbiol. 2025 Sep 3;16:1648467. doi: 10.3389/fmicb.2025.1648467 (PMC12440919; doi:10.3389/fmicb.2025.1648467)
Supplement: Supplementary file 1 [file Data_Sheet_1.PDF]

Supplementary Material

Supplementary Table 1. Gene-Specific Primer Sequences for RT-qPCR

Primer name	Sequence (5'-3')	Product Length
KAR2-F	CTGAAGATTACCTTGGCAAAAAAGT	85bp
KAR2-R	TTAGTAGCTTGTCTTTGAGCATCGTT	
SEC61-F	GTCACAGAGACACTTCTGCTTACAA	132bp
SEC61-R	TAGACGTACCAGAACCAAGAGTACC	
YSY6-F	ACACCTAAACAAAGAGCAGCTAATG	148bp
YSY6-R	TTGCTCCACCACATACTAAGAA	
HWP1-F	AGGTAGACGGTCAAGGTGAAACAG	179bp
HWP1-R	TGGCTCTTGTGGTTGTTGTTGTGTG	
ALS1-F	GCAAACCCAGGAGACACATTCAC	93bp
ALS1-R	AACACCGTCAGCAGTCAAATCAAC	
ALS3-F	CCGGTTTCATCTGAATCATTTAGTT	145bp
ALS3-R	ACGACAAGGTGTACGAATTAACATCT	
HGC1-F	AACCACCACCACCAATGAA	166bp
HGC1-R	GAAACAGCACGAGAACCAG	
CYR1-F	TGAGCCACCAATAGGAC	174bp
CYR1-R	AACGCATCACCTTCAGT	
ACT1-F	GAAGCCCAATCCAAAAGA	130bp
ACT1-R	CTTCTGGAGCAACTCTCAATTC	

Supplementary Table 2. Fecal fungal burden in mice from the SN152 and Ire1Δ/Δ groups in the gastrointestinal colonization model.

Day(d)	SN152 (Log_{10}CFU±SD)	Ire1Δ/Δ (Log_{10}CFU±SD)
1	7.490±0.164	6.430±0.162
2	7.415±0.231	6.216±0.106
3	7.220±0.11	5.039±0.300
4	6.918±0.232	4.105±0.593
5	6.929±0.061	3.461±0.547
6	6.874±0.112	0±0
7	6.907±0.304	0±0

Supplementary Table 3. Fungal burden in gastrointestinal tissues of mice from the SN152 and Ire1Δ/Δ groups in the gastrointestinal colonization model.

Tissue	SN152(Log_{10}CFU±SD)	Ire1Δ/Δ(Log_{10}CFU±SD)
stomach	5.780±0.292	0±0
s.intest	5.132±0.298	0±0
caecum	5.953±0.247	0±0
l.intest	5.994±0.136	0±0

Supplementary Table 4. Fecal fungal burden in mice from the SC5314 and 4μ8c treatment groups in the gastrointestinal colonization model.

Day(d)	SC5314 (Log_{10}CFU±SD)	4μ8c (Log_{10}CFU±SD)
1	7.706±0.097	7.082±0.183
2	7.525±0.158	6.511±0.131
3	7.415±0.2	6.338±0.092
4	7.343±0.174	6.242±0.147
5	7.051±0.153	6.187±0.225
6	6.757±0.214	6.095±0.32
7	6.722±0.301	6.092±0.292

Supplementary Table 5. Fungal burden in gastrointestinal tissues of mice from the SC5314 and 4μ8c treatment groups in the gastrointestinal colonization model.

Tissue	SC5314 (Log_{10}CFU±SD)	4μ8c (Log_{10}CFU±SD)
stomach	6.604±0.165	6.033±0.145
s.intest	5.715±0.134	5.282±0.109
caecum	6.458±0.135	5.933±0.215
l.intest	6.843±0.247	5.938±0.311

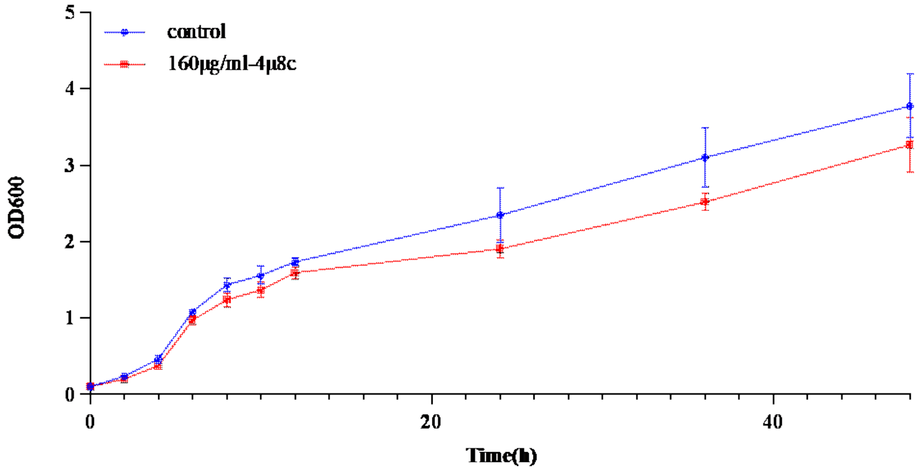

Supplementary Figure 1. Effect of 4μ8c on the growth ability of *C. albicans*. The growth curves of *C. albicans* in the control and 4μ8c treatment groups were measured at 0 h, 2 h, 4 h, 6 h, 8 h, 10 h, 12 h, 24 h, 32 h, and 48 h using a microplate reader to determine the OD_{600} values. The results show no statistically significant difference between the growth curves of the control and treatment groups ($P > 0.05$).

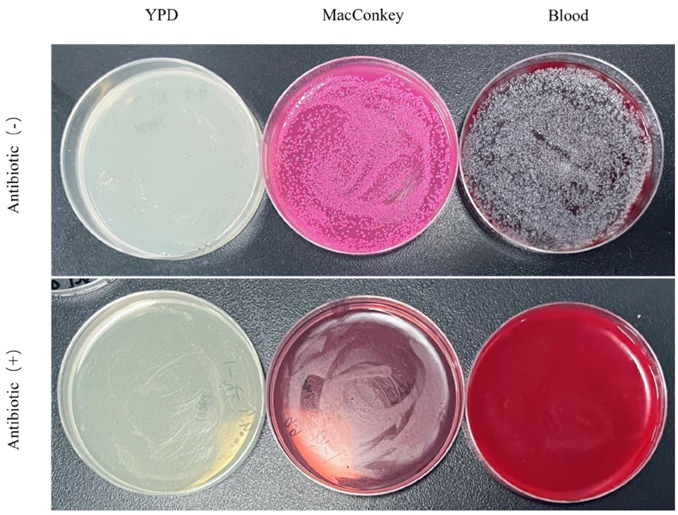

Supplementary Figure 2. Comparison of fecal fungal burden in mice with or without antibiotic treatment. Verification of gut microbiota depletion: Fecal samples from antibiotic-treated and untreated groups were collected on day 4 post-pretreatment, weighed, homogenized in sterile PBS, and plated on YPD agar (supplemented with 100 µg/mL ampicillin, 50 µg/mL kanamycin, and 100 µg/mL streptomycin), MacConkey agar, or blood agar to assess bacterial growth.

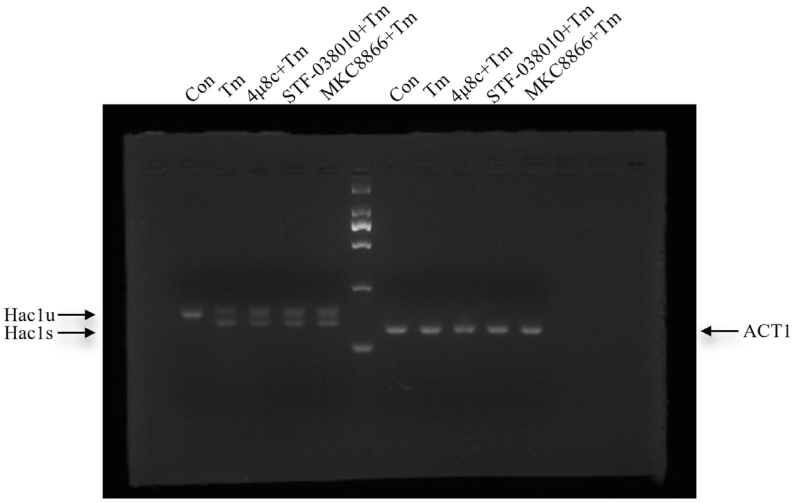

Supplementary Figure 3. Uncropped original gel image for qualitative analysis of HAC1 mRNA expression in *C. albicans* (Figure 2A).

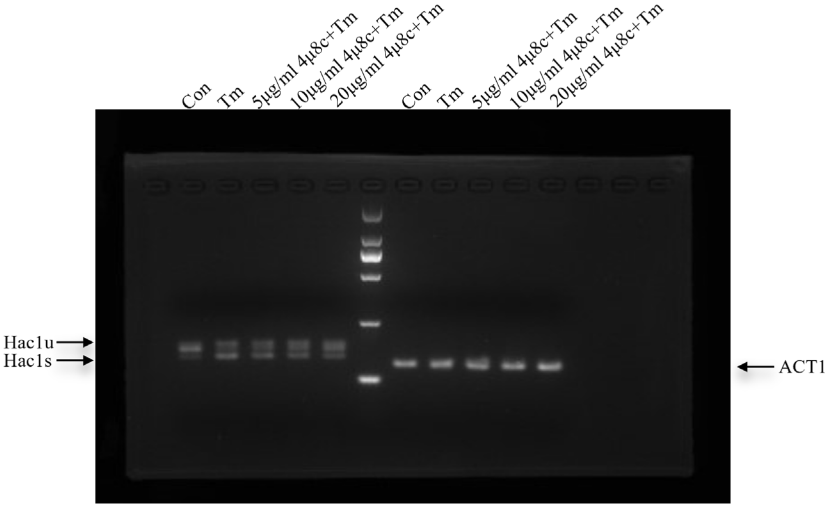

Supplementary Figure 4. Uncropped original gel image for qualitative analysis of HAC1 mRNA expression in *C. albicans* (Figure 2C).

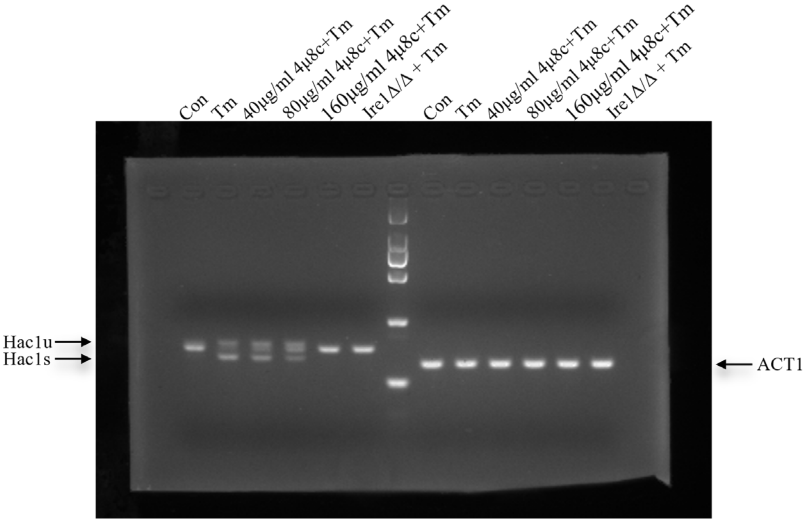

Supplementary Figure 5. Uncropped original gel image for qualitative analysis of HAC1 mRNA expression in *C. albicans* (Figure 2D).

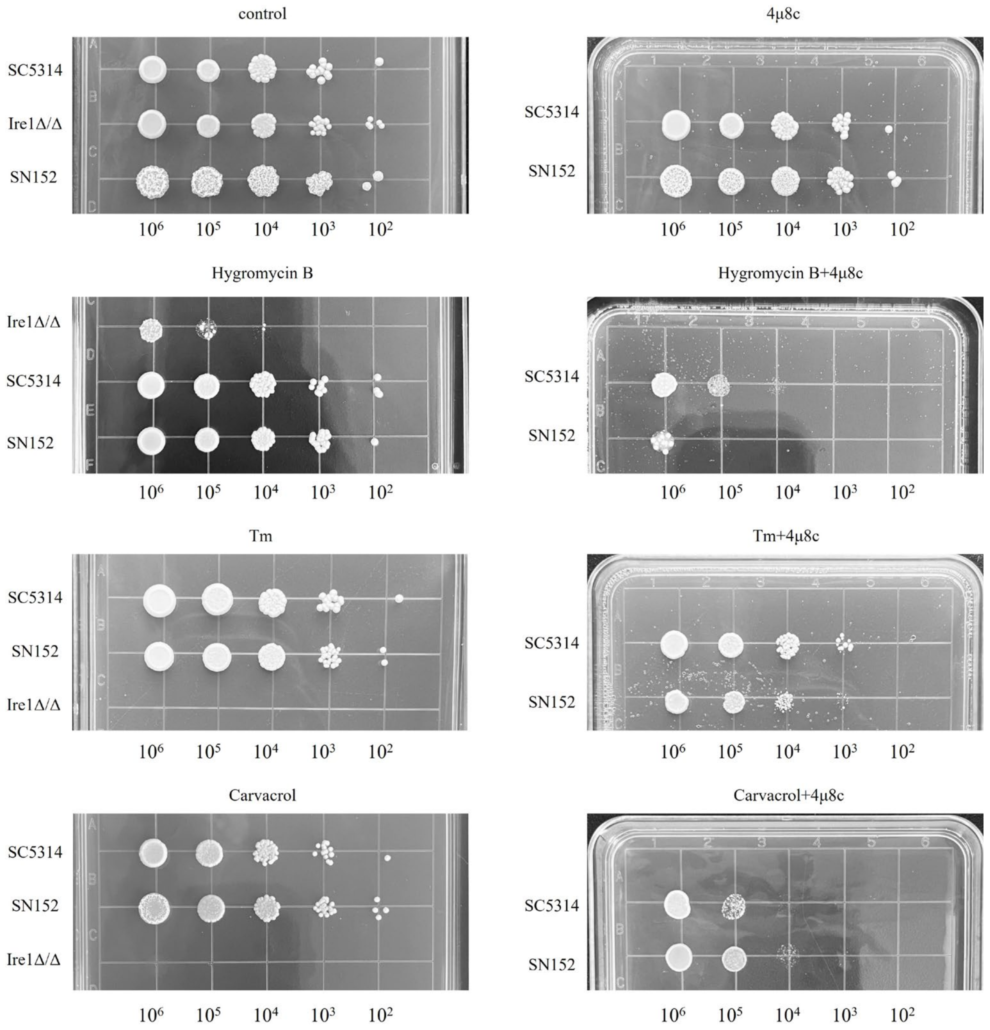

Supplementary Figure 6. Uncropped original image showing the growth of *C. albicans* cultures (SC5314, Ire1Δ/Δ strain, and the parental strain SN152 of the Ire1Δ/Δ strain) on YPD solid plates with or without hygromycin B, carvacrol, Tm, ITZ and FLU in the presence or absence of 4μ8c (Figure 6).

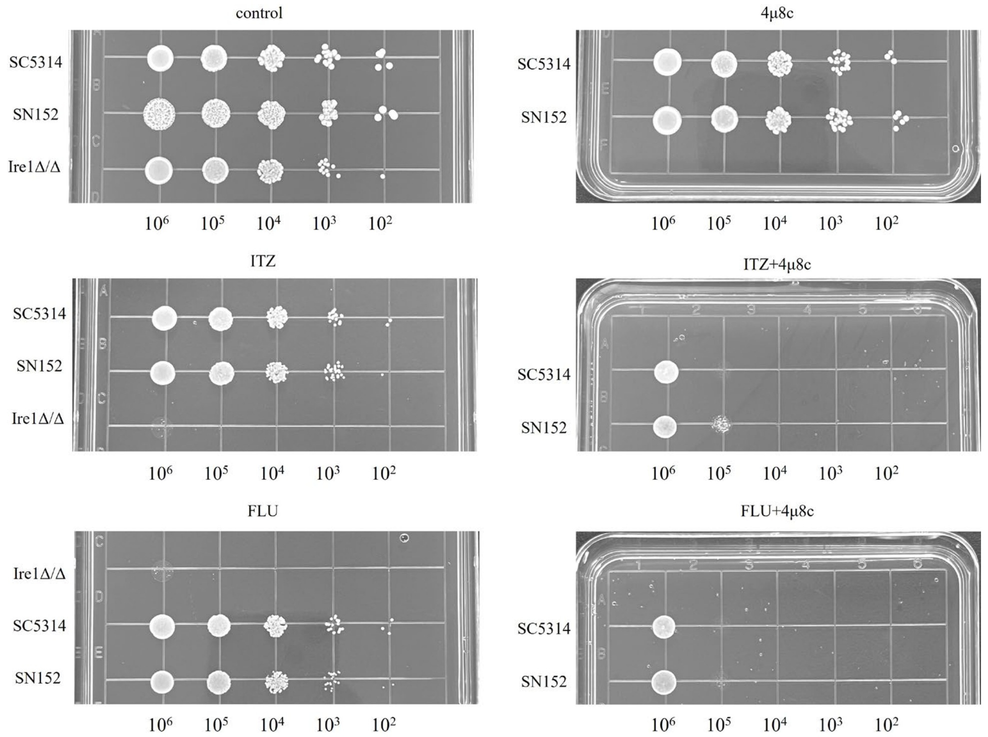

Supplementary Figure 7. Uncropped original image showing the growth of *C. albicans* cultures (SC5314, Ire1Δ/Δ strain, and the parental strain SN152 of the Ire1Δ/Δ strain) on YPD solid plates with or without ITZ and FLU, in the presence or absence of 4μ8c (Figure 6B).
